# Metastasis, Risk Factors and Prognostic Significance of Splenic Hilar Lymph Nodes in Gastric Adenocarcinoma

**DOI:** 10.1371/journal.pone.0099650

**Published:** 2014-06-10

**Authors:** Xiao-Long Chen, Kun Yang, Wei-Han Zhang, Xin-Zu Chen, Bo Zhang, Zhi-Xin Chen, Jia-Ping Chen, Zong-Guang Zhou, Jian-Kun Hu

**Affiliations:** Department of Gastrointestinal Surgery, West China Hospital, Sichuan University, Sicuhan, China; University of Pisa, Italy

## Abstract

**Background:**

The metastatic rate and risk factors of splenic hilar (No.10) lymph nodes (LNs) in gastric adenocarcinoma were still variable and uncertain, and the prognostic significance of No.10 LNs was also controversial. The aim of this retrospective study was to analyze the metastatic rate, risk factors and prognostic significance of No.10 LNs in gastric adenocarcinoma.

**Methods:**

From August 2007 to December 2011, 205 patients who were diagnosed with primary gastric adenocarcinoma and underwent total or proximal gastrectomy plus No.10 LNs dissection in West China Hospital were enrolled. Clinicopathological features and survival outcomes were retrospectively analyzed.

**Results:**

Mean numbers of harvested LNs and metastatic LNs were 34.8±12.6 (15–73) and 8.7±10.8 (0–67), respectively. The proportion of cases with positive No.10 LNs was 8.8% (18/205). In all 204 dissected No.10 LNs, 47 LNs (23.0%) were metastatic. In 52.2% (107/205) patients, the dissected splenic hilar tissues were histologically determined as only fat tissues but without LNs structure. Histological evidence of LNs structure was found in 98 (47.8%) patients with 18.4% (18/98) metastatic No.10 LNs. In multivariate logistic regression analysis, metastasis of No.10 LNs was significantly correlated with No.4sa LNs (p = 0.010) and pN stage (p = 0.012). Regarding survival analysis, 199 (97.1%) patients were followed up (0.6–74.8 months). In all patients with R0 resection, metastatic No.10 LNs caused significantly worse prognosis both in Kaplan-Meier (p = 0.006) and Cox regression analysis (p = 0.031).

**Conclusions:**

Although the metastatic rate of No.10 LNs was 8.8%, dissection of No.10 LNs might be meaningful due to the poor prognosis of positive cases. And attentions should be also paid to its correlated factors including pN stage and No.4sa LNs.

## Introduction

Gastric cancer (GC) is one of the most common malignancies worldwide with high incident rate and cancer-related mortality, especially in Asia [1]–[3]. At present, surgery is considered as the principal treatment for resectable GC. Radical gastrectomy plus lymphadenectomy is the important development in GC treatment. As we know, lymphatic metastasis is the principal and common metastatic pattern of GC. It is important to dissect enough extent of regional lymph nodes (LNs) to achieve the radical effect in order to improve postoperative survival outcomes. Dutch trial compared the long-term oncological effectiveness between D1 and D2 lymphadenectomy for GC and showed lower locoregional recurrence and GC-related mortality in D2 group than in D1 group [4]. Additionally, it was reported that survival benefits of D2 plus para-aortic lymphadenectomy was not significantly greater than those of D2 lymphadenectomy alone, but might cause more complications partly [5]–[6]. Therefore, to date, it is widely accepted that D2 lymphadenectomy is important and necessary for advanced GC, especially in Asia.

Nowadays, the incidence of proximal GC is gradually increasing [7]. Total and proximal gastrectomies are both indicated for proximal GC [8]. In total gastrectomy, splenic hilar (No.10) LNs are required to be dissected in D2 lymphadenectomy range according to Japanese gastric cancer treatment guideline 2010 (version 3) by Japanese Gastric Cancer Association (JGCA), but not required in proximal gastrectomy [9]. Metastatic rates of No.10 LNs were previously reported from 7.3%–30% in proximal GC [10]–[17]. Some studies reported that metastatic No.10 LNs were closely associated with worse prognosis than negative ones [14], [17]–[20], but other report showed a different result [21]. Besides, No.10 LNs are also correlated with clinicopathological features and other regional LNs, but the relationship is still uncertain.

Because of the variable metastatic rate of No.10 LNs and the adjacent anatomical relationship between No.10 LNs and spleen, it seems that gastrectomy and splenectomy should be combined theoretically for proximal GC to fulfill curative intention [22]–[23]. However, many reports showed no benefits from routine or prophylactic splenectomy, compared with spleen preservation, but contrarily splenectomy caused more postoperative complications [8], [18], . Thus splenectomy remains controversial in GC surgery. For this reason, it is necessary to analyze the prognostic significance of metastatic No.10 LNs in GC. Hence the aim of this study was to retrospectively research the metastatic status and prognostic significance of No.10 LNs in GC.

## Methods

This retrospective study was approved by Ethics Committee of West China Hospital, Sichuan University. Written informed consent was not obtained but patient records were anonymized and de-identified prior to analysis.

### Inclusion criteria

We retrospectively enrolled the patients from August 2007 to December 2011, who were histologically diagnosed with GC and underwent total or proximal gastrectomy with No.10 LNs dissection in Department of Gastrointestinal Surgery, West China Hospital, Sichuan University. All the patients were neo-adjuvant chemotherapy-naïve, and adjuvant chemotherapy or chemoradiotherapy were given according to patients' personal willing. There was no limitation of age, sex or race.

### Surgery

For middle and upper thirds tumors, total gastrectomy was preferred, while some selected cardia tumors (early disease or small-sized) were considered for proximal gastrectomy with negative margins by intra-operative frozen section. All the patients received D2/D2+ lymphadenectomy according to the classification of JGCA. Synchronous prophylactic splenectomy was not routinely performed. Due to the complicated structure of splenic artery and vein in hilar region, enbloc resection of No. 10 LNs was sometimes not able to be achieved in primary lymphadenectomy for the sake of surgical safety. After stomach was removed, secondary lymphadenectomy of No. 10 LNs including all the fat, lymphatic and connective tissues along distal splenic artery and splenic hilum was performed by ultrasonic scalpel.

### Data collection and follow up

Clinicopathological characteristics, mainly concerning macroscopic type, differentiation grade, tumor location, maximal diameter of tumor and pathological TNM stage according to Japanese classification of gastric carcinoma (3^rd^ English version) by JGCA [28] were recorded. Follow-up through telephones, mails and outpatient visit were conducted up to December 2013.

### Statistical analysis

Statistical analysis was performed by statistical software SPSS 17.0. The risk factors of No.10 LNs metastasis were analyzed by Chi-square test, rank sum test and student's t-test for univariate analysis and logistic regression for multivariate analysis. Kaplan-Meier and life table method were used to calculate the cumulative survival rate. Log-rank test and Cox regression were conducted to accomplish univariate and multivariate survival analyses. Two-sided P value less than 0.05 was considered as statistical significance.

## Results

### Patients

Two hundred and five patients were diagnosed with GC, in whom 46 (22.4%) and 159 (77.6%) patients underwent proximal and total gastrectomy respectively. Average operation time was 266.0±42.3 min. Open surgeries were carried out for 145 patients and laparoscopy-assisted surgery for 60 patients, in whom 2 patients received conversion to laparotomy. Surgeons decided whether to perform combined resection of other organs according to the situation of GC invasion and other independent diseases. Finally, fourteen (6.8%) patients underwent gastrectomy combined with other organs resections, including three splenectomies, three hepatic nodules resections, three gallbladder resections, two resections for part of diaphragm, and four resections for part of small intestine. Among them, one patient underwent both hepatic nodule and part of diaphragm resection. After pathological examination of these resected organs, the dissected hepatic nodule in one patient and the diaphragm in another patient were confirmed as carcinoma cells invasion (T4b).

### Metastasis of No.10 LNs

**Figure 1:**
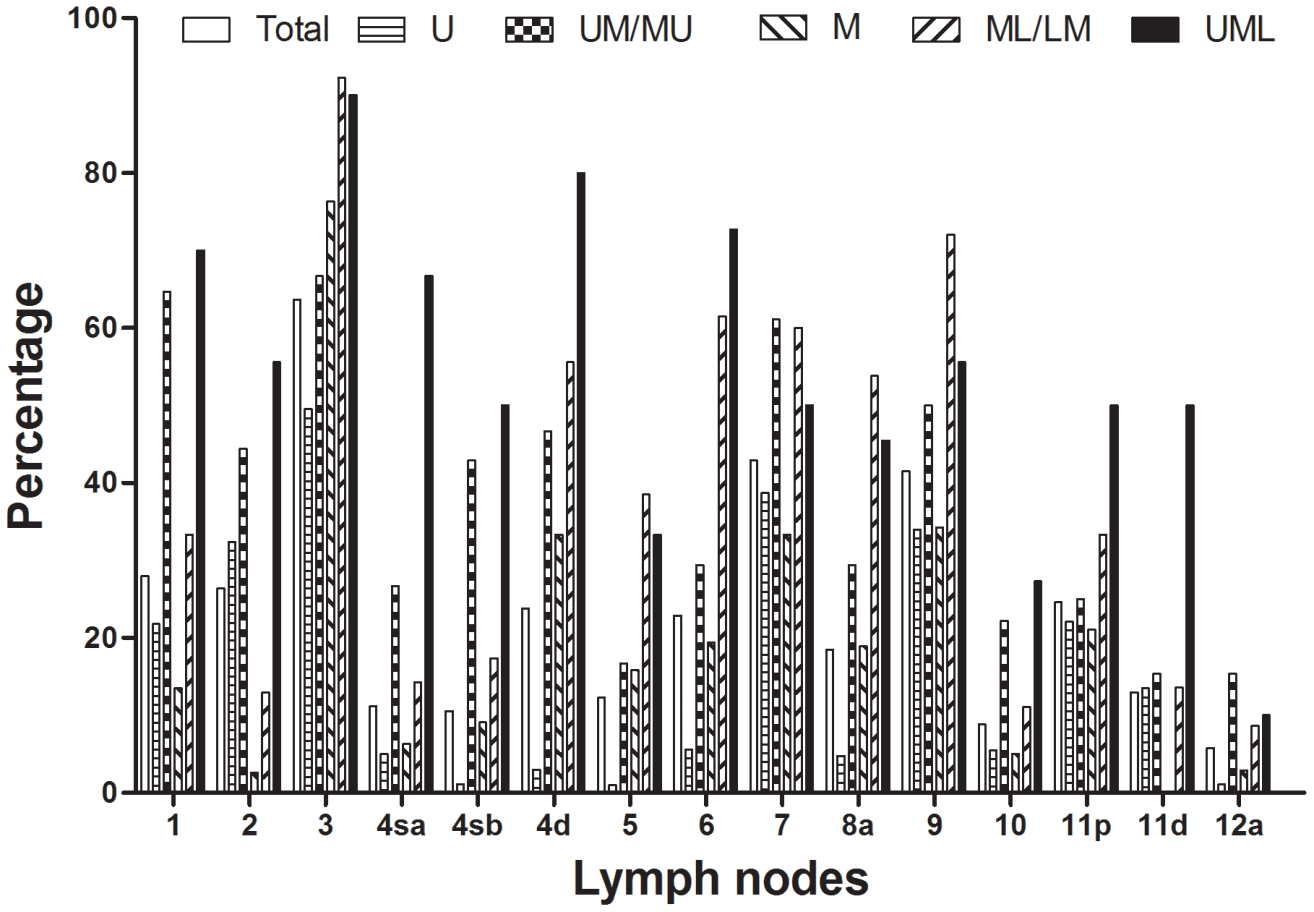
Metastatic rates of harvested lymph nodes according to tumor locations.

Average numbers of total dissected LNs was 34.8±12.6 (15–73) and metastatic LNs was 8.7±10.8 (0–67) in all patients. The metastatic rates of No.10 LNs were 8.8% (18/205) in all patients, 6.5% (3/46) in proximal gastrectomy and 9.4% (15/159) in total gastrectomy. In total 204 dissected No.10 LNs, 47 (23.0%) metastatic No.10 LNs were found. Histological evidence of dissected No.10 LNs was found in 98 patients (47.8%, 98/205), while the dissected No.10 LNs were determined as fat tissues in 107 patients (52.2%, 107/205). For the 98 cases with histological evidence of No.10 LNs, the metastatic rates were 18 (18.4%) in all patients, 14.3% (3/21) in proximal gastrectomy and 19.5% (15/77) in total gastrectomy. The metastatic rates of regional LNs within D2 range were illustrated according to tumor longitudinal location in Figure 1. The highest metastatic rates were No.3 LNs (63.7%), No.7 LNs (42.9%) and No.9 LNs (41.5%). Metastatic rate of No.10 LNs (8.8%) was located at relative lower level among all LNs. According to the longitudinal location of tumors, the metastatic rates of No.10 LNs at U, UM/MU, M, ML/LM and UML were 5.5%, 22.2%, 5.0%, 11.1% and 27.3% respectively in all patients, and 13.0%, 50.0%, 8.3%, 25.0% and 37.5% respectively in 98 patients with histological evidence of dissected No.10 LNs. With respect to the cross-sectional location of GC, the metastatic rates of No.10 LNs were 3.8%, 18.2%, 0.0%, 13.3%, and 13.3% at lesser, greater curvature, anterior, posterior wall and multi-walls involved respectively in all patients, and 8.2%, 40.0%, 0%, 28.6% and 25.8% respectively in 98 patients with histological evidence of dissected No.10 LNs.

### Clinicopathological characteristics relationship

Demographic and correlations between No.10 LNs and clinicopathological characteristics were shown in Table 1 and Table 2. Univariate analysis showed longitudinal location (p = 0.022), cross-sectional location (p = 0.047), tumor size (p = 0.001), pT stage (p = 0.041), pN stage (p<0.001), M stage (p = 0.003) and pTNM stage (p<0.001) were significantly correlated with metastasis of No.10 LNs. The percentages showed that carcinomas at UM/MU and UML, greater curvature, posterior and multi-walls involved of stomach, the maximal diameter of tumors larger than 5 cm, T4b, pN3, M1 and pTNM IIIC-IV stages were obviously associated with positive No.10 LNs. Besides, univariate analysis revealed that the metastasis of No.10 LNs was remarkably relevant to No.1 LNs (p<0.001), No.2 LNs (p = 0.001), No.3 LNs (p = 0.004), No.4sb LNs (p = 0.018), No.4sa LNs (p<0.001), No.4d LNs (P = 0.002), No.6 LNs (P<0.001), No.7 LNs (P<0.001), No.9 LNs (P<0.001), No.11d LNs (p = 0.018), No.12a LNs (p = 0.008) respectively. However, differentiation grade (p = 0.375), macroscopic type (p = 0.087), No.5 LNs (P = 0.094), No.8a LNs (p = 0.094) and No.11p LNs (p = 0.172) were not notably in relation to the metastasis of No.10 LNs. In logistic regression analysis, No.4sa LNs (p = 0.010) and pN stage (p = 0.012) were significantly correlated with the metastasis of No.10 LNs.

**Table 1:** Details of clinicopathological characteristics and univariate correlation analysis of No.10 LNs.

	Characteristics	Group A	Group B	Group C	P value	P value
		No.10 LNs (+)	No.10 LNs (-)	No.10 LNs (-)	A vs B	A vs C
		n = 18 (%)	n = 187 (%)	80 (%)		
Age	Mean	56.0±10.8	58.7±10.3	58.1±10.1	0.294*	0.434*
	≥60yrs	6 (33.3)	87 (46.5)	34 (42.5)	0.283	0.475
	<60yrs	12 (66.7)	100 (53.5)	46 (57.5)		
Gender	Male	13 (72.2)	138 (73.8)	57 (71.3)	1.000	0.934
	Female	5 (27.8)	49 (26.2)	23 (28.7)		
Longitudinal	U	6 (33.3)	103 (55.1)	40 (50.0)	0.022	0.033
location	UM/MU	4 (22.2)	14 (7.5)	4 (5.0)		
	M	2 (11.1)	38 (20.3)	22 (27.5)		
	ML/LM	3 (16.7)	24 (12.8)	9 (11.3)		
	UML	3 (16.7)	8 (4.3)	5 (6.2)		
Cross-sectional	Lesser curvature	4 (22.2)	102 (54.5)	45 (56.2)	0.047	0.035
location	Greater curvature	4 (22.2)	18 (9.6)	6 (7.5)		
	Anterior	0 (0)	2 (1.1)	1 (1.3)		
	Posterior	2 (11.1)	13 (7.0)	5 (6.2)		
	Multi-walls involved	8 (44.4)	52 (27.8)	23 (28.8)		
Differentiation	Well	0 (0)	0 (0)	0 (0)	0.375	0.729
grade	Moderate	2 (11.1)	41 (21.9)	14 (17.5)		
	Poor	16 (88.9)	146 (78.1)	66 (82.5)		
Macroscopic	Type 0	0 (0)	10 (5.3)	5 (6.2)	0.087	0.232
type	Type 1	0 (0)	15 (8.0)	9 (11.3)		
	Type 2	11 (61.1)	94 (50.3)	40 (50.0)		
	Type 3	2 (11.1)	50 (26.7)	16 (20.0)		
	Type 4	5 (27.8)	18 (9.6)	10 (12.5)		
Tumor size	≤2 cm	0 (0)	13 (7.0)	4 (5.0)	0.001	0.009
	2–5 cm	2 (11.1)	77 (41.2)	28 (35.0)		
	5–8 cm	6 (33.3)	67 (35.8)	34 (42.5)		
	>8 cm	10 (55.6)	30 (16.0)	14 (17.5)		

Abbreviation: *: Student t test; LNs: lymph nodes. Group A: positive No.10 LNs; Group B: all negative No.10 LNs; Group C: negative No.10 LNs with histological evidence LNs structure.

**Table 2:** Details of pathological stage and univariate correlation analysis of No.10 LNs.

	Characteristics	Group A	Group B	Group C	P value	P value
		No.10 LNs(+)	No.10 LNs (−)	No.10 LNs (−)	A vs B	A vs C
		n = 18 (%)	n = 187 (%)	80 (%)		
T stage	T1	1 (5.6)	11 (58.8)	5 (6.2)	0.041	0.085
	T2	0 (0)	20 (10.7)	7 (8.8)		
	T3	1 (5.6)	21 (11.2)	7 (8.8)		
	T4a	8 (44.4)	107 (57.2)	49 (61.2)		
	T4b	8 (44.4)	28 (15.0)	12 (15.0)		
N stage	N0	0 (0)	45 (24.1)	18 (22.5)	<0.001	0.001
	N1	0 (0)	33 (17.6)	10 (12.5)		
	N2	1 (5.6)	38 (20.3)	18 (22.5)		
	N3	17 (94.4)	71 (38.0)	34 (42.5)		
M stage	M0	10 (55.6)	161 (86.1)	70 (87.5)	0.003	0.004
	M1	8 (44.4)	26 (13.9)	10 (12.5)		
TNM stage	I	0 (0)	22 (11.8)	8 (10.0)	<0.001	0.002
	II	1 (5.6)	33 (17.6)	12 (15.0)		
	IIIA	0 (0)	25 (13.4)	10 (12.5)		
	IIIB	0 (0)	34 (18.2)	17 (21.2)		
	IIIC	9 (50.0)	47 (25.1)	23 (28.8)		
	IV	8 (44.4)	26 (13.9)	10 (12.5)		

Abbreviation: LNs: lymph nodes; Group A: positive No.10 LNs; Group B: all negative No.10 LNs; Group C: negative No.10 LNs with histological evidence LNs structure.

For the group of patients with histological evidence of LNs structure in No.10 LNs, the correlated factors were also analyzed. Similarly, longitudinal location (p = 0.033), cross-sectional location (p = 0.035), tumor size (p = 0.009), pN stage (p = 0.001), M stage (p = 0.004) and pTNM stage (p = 0.002) were also significantly correlated with metastasis of No.10 LNs (Table 1, Table 2). With respect to regional LNs, No.1 LNs (p<0.001), No.2 LNs (p = 0.001), No.3 LNs (p = 0.008), No.4sb LNs (p = 0.024), No.4sa LNs (p<0.001), No.4d LNs (P = 0.011), No.6 LNs (P<0.001), No.7 LNs (P<0.001), No.9 LNs (P<0.001), No.11d LNs (p = 0.017), No.12a LNs (p = 0.012) were obviously associated with metastasis of No.10 LNs. However, differentiation grade (p = 0.729), macroscopic type (p = 0.232), pT stage (p = 0.085), No.5 LNs (P = 0.255), No.8a LNs (p = 0.188) and No.11p LNs (p = 0.125) were not notably relevant to the metastasis of No.10 LNs. For multivariate analysis, pN stage (p = 0.018) and No.4a (p = 0.022) were remarkably correlated with positive No.10 LNs cases.

### Survival

**Figure 2:**
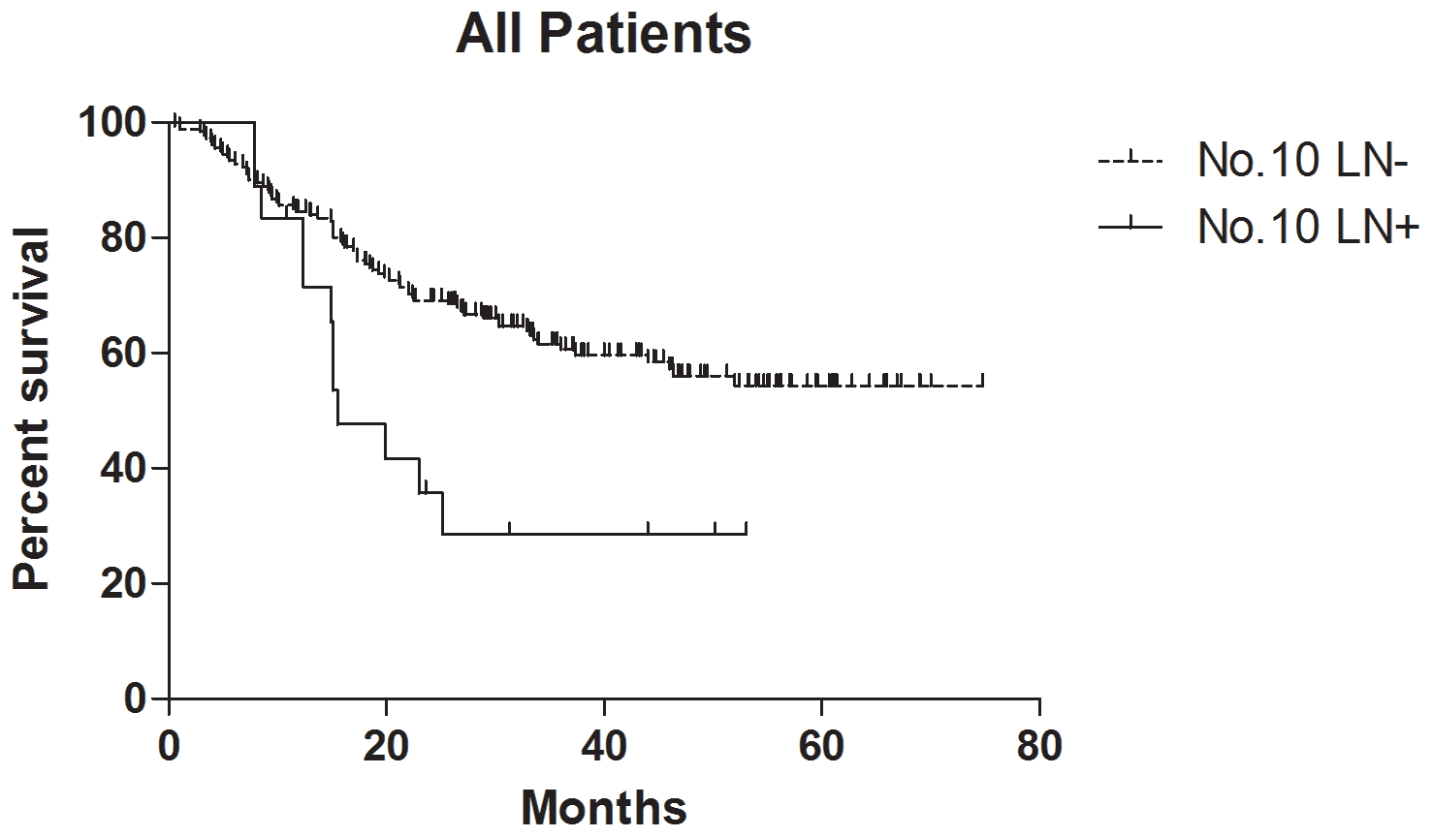
Kaplan-Meier survival analysis of No.10 LNs for all patients.

**Figure 3:**
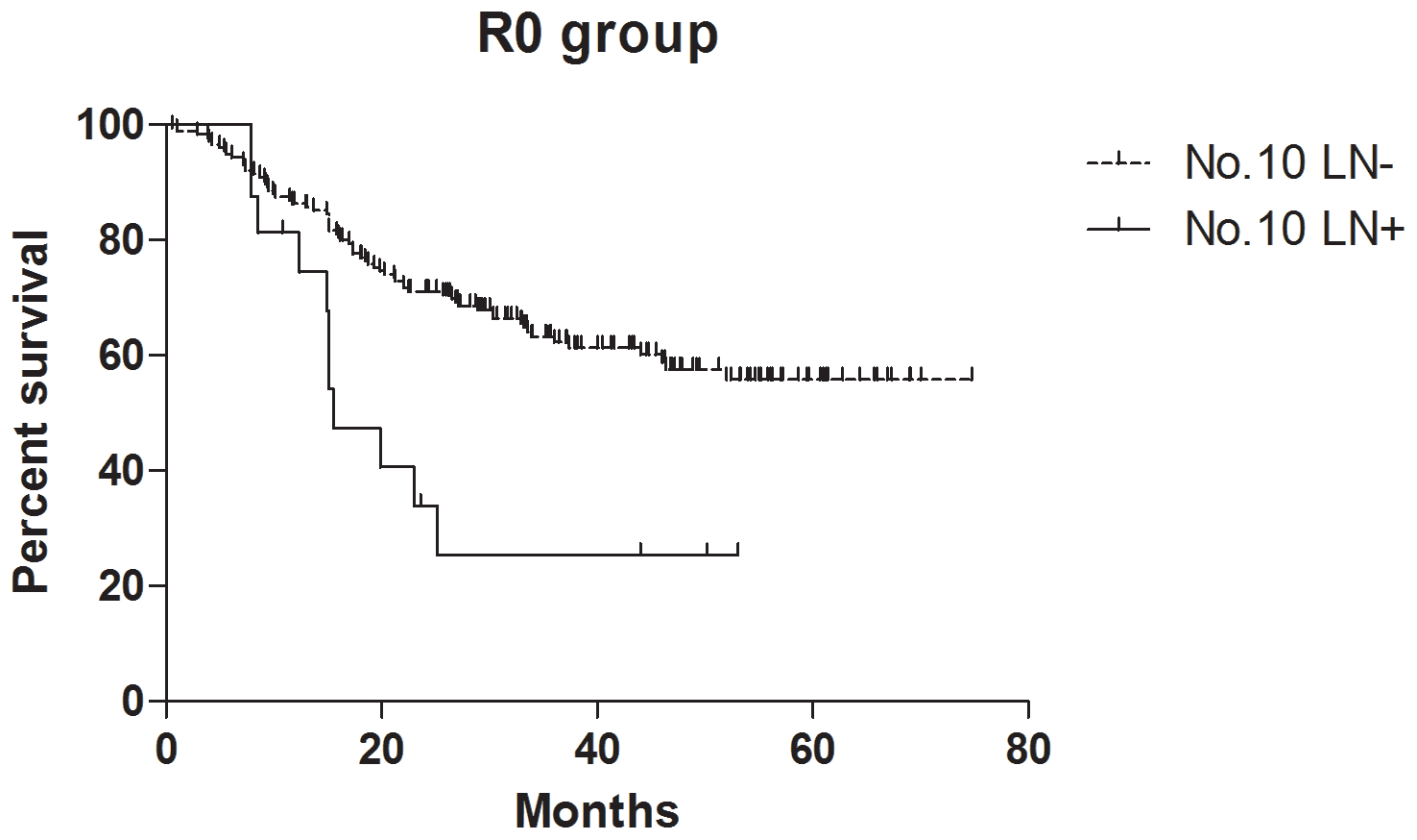
Kaplan-Meier survival analysis of No.10 LNs for R0 group in all patients.

**Figure 4:**
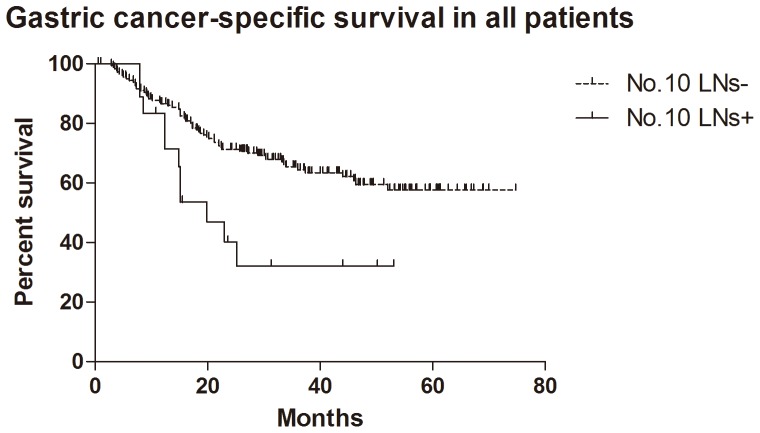
Kaplan-Meier survival analysis of gastric cancer specific group in all patients

In total 205 patients, 199 patients (199/205, 97.1%) were followed up for survival analysis with median 29.9 (0.6–74.8) months. Because of the limitation of follow-up time, the survival rate was larger than 50% and the median survival time was not figured out in the group with negative No.10 LNs. The median survival time of positive No.10 LNs was 15.9, 19.5 and 15.9 months in all patients, GC specific group and R0 resection group respectively. The 1- and 2-year cumulative survival rates of overall survival, GC specific group, R0 resection group and GC specific + R0 resection group were 83% and 66%, 85% and 69%, 85% and 68%, 87% and 71% respectively (Table 3). The comparison of cumulative survival rates of negative and positive No.10 LNs cases were also shown in Table 3. Kaplan-Meier analysis showed that the differences between negative and positive No.10 LNs were significant in overall survival (n = 199, p = 0.006, HR = 0.30, 95%CI [0.13–0.71], Figure 2), R0 resection group (n = 191, p = 0.003, HR = 0.18, 95%CI [0.07–0.47],Figure 3) and GC specific group (n = 199, p = 0.007, HR = 0.29, 95%CI [0.12–0.71], Figure 4). In Cox regression analysis, we found No.10 LNs was not an independent prognostic factor in all patients, but an independent prognostic factor in R0 resection group (p = 0.031), in addition to pTNM stage (p = 0.001) and longitudinal location (p = 0.005).

**Table 3:** Survival outcomes after surgery and Kaplan-Meier univariate analysis.

		N (%)	Median survival	Survival rate (%)		P	HR
			time (month)	1-year	2-year	value	95%CI
All patients							
Overall survival		199(100.0)	—	83%	66%	0.006	0.30(0.13–0.71) 0.7128)
No.10 LNs	Negative	181 (91.0)	—	84%	69%		
	Positive	18 (9.0)	15.9	71%	35%		
Gastric cancer specific		199(100.0)	—	85%	69%	0.007	0.29(0.12–0.71)
No.10 LNs	Negative	181 (91.0)	—	86%	71%		
	Positive	18 (9.0)	19.5	71%	39%		
R0 resection		191(96.0)	—	85%	68%	0.003	0.18(0.07–0.47)
No.10 LNs	Negative	175 (87.9)	—	86%	71%		
	Positive	16 (8.0)	15.9	74%	33%		
Gastric cancer specific +R0 resection		191(96.0)	—	87%	71%	0.003	0.22(0.079–0.59)
No.10 LNs	Negative	175 (87.9)	—	88%	73%		
	Positive	16 (8.0)	19.5	74%	37%		
Patients with histological evidence of LNs structure in No.10 LNshistologicalevidenceof LNs structure.structure							
Overall survival		94(100.0)	—	81%	62%	0.017	0.36(0.15–0.83)
No.10 LNs	Negative	76 (80.9)	—	83%	68%		
	Positive	18 (19.1)	15.9	71%	35%		
Gastric cancer specific		94(100.0)	—	83%	65%	0.015	0.33(0.13–0.80)
No.10 LNs	Negative	76 (80.9)	—	85%	71%		
	Positive	18 (19.1)	19.5	71%	39%		
R0 resection		89 (94.7)	—	81%	62%	0.010	0.30(0.12–0.74)
No.10 LNs	Negative	73 (77.7)	—	84%	70%		
	Positive	16 (17.0)	15.9	74%	33%		
Gastric cancer specific +R0 resection		89 (94.7)	—	85%	67%	0.009	0.27(0.10–0.72)
No.10 LNs	Negative	73(77.7)	—	88%	73%		
	Positive	16 (17.0)	19.5	74%	37%		

Abbreviation: LNs: lymph nodes.

**Figure 5:**
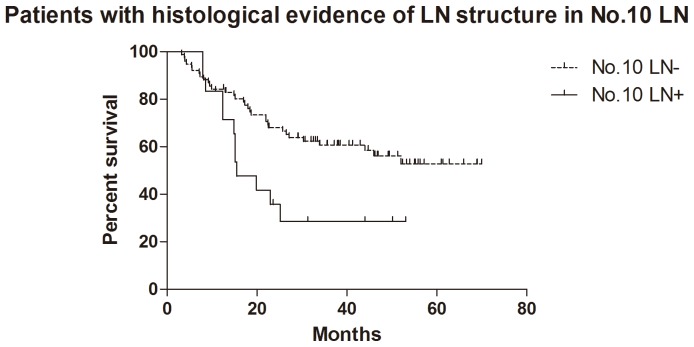
Kaplan-Meier survival analysis of No.10 LNs for patients with histological evidence of LNs structure in No.10 LNs.

**Figure 6:**
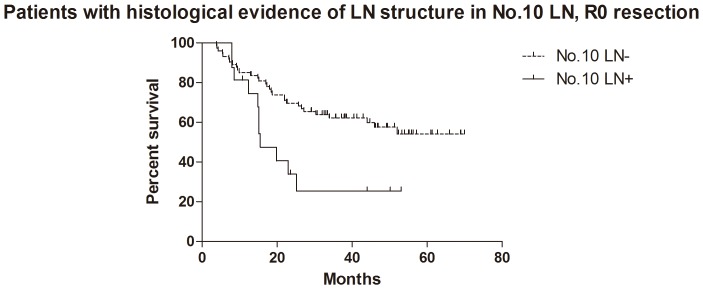
Kaplan-Meier survival analysis of No.10 LNs for R0 group in patients with histological evidence of LNs structure in No.10 LNs.

**Figure 7:**
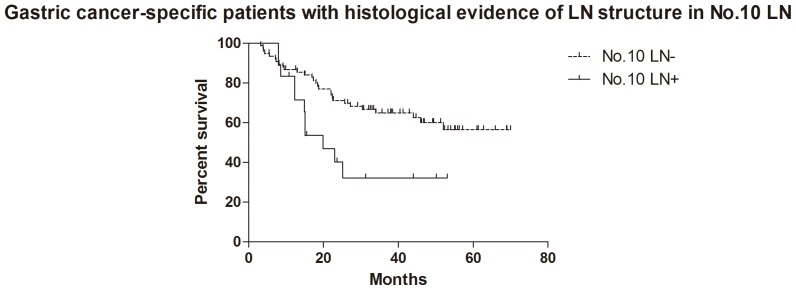
Kaplan-Meier survival analysis of gastric cancer specific group in patients with histological evidence of LNs structure in No.10 LNs.

**Figure 8:**
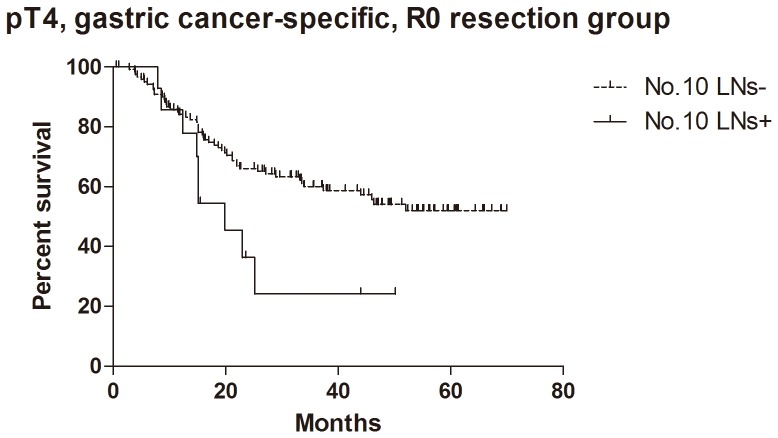
Kaplan-Meier survival analysis of pT4 stage gastric cancer specific + R0 resection group in all patients.

**Figure 9:**
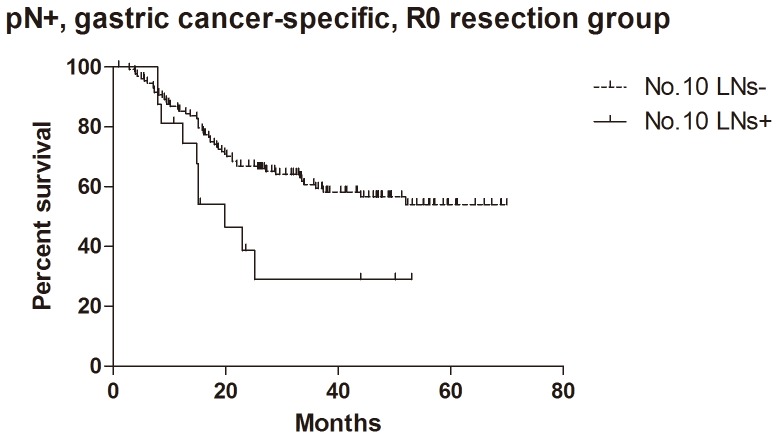
Kaplan-Meier survival analysis of pN+ stage gastric cancer specific + R0 resection group in all patients.

**Figure 10:**
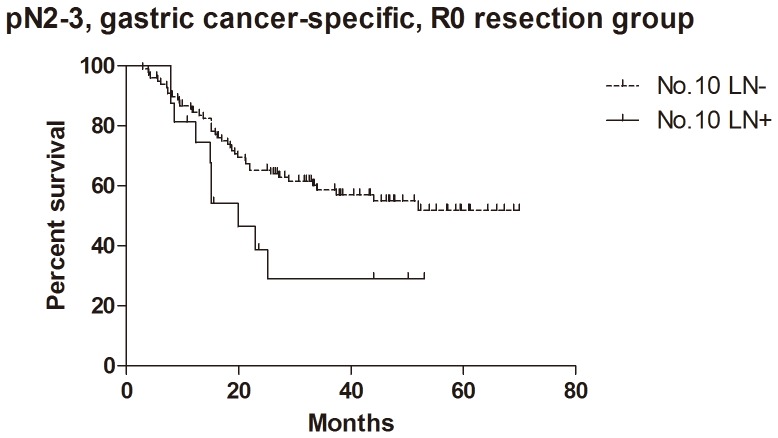
Kaplan-Meier survival analysis of pN2-3 stage gastric cancer specific + R0 resection group in all patients.

**Figure 11:**
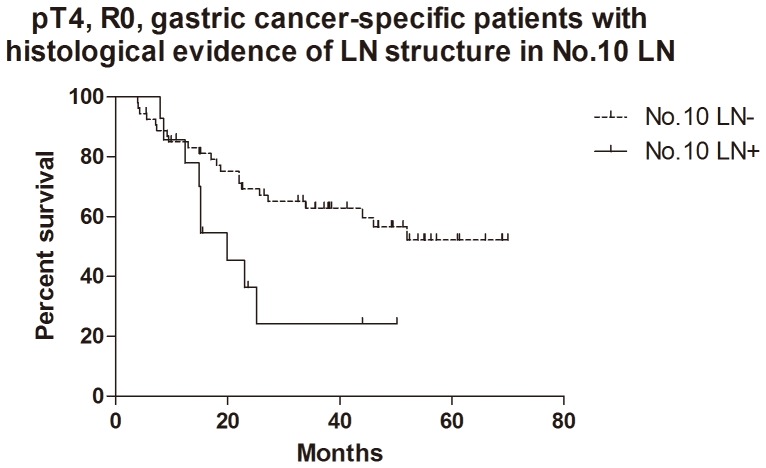
Kaplan-Meier survival analysis of pT4 stage gastric cancer specific + R0 resection group in patients with histological evidence of LNs structure in No.10 LNs.

**Figure 12:**
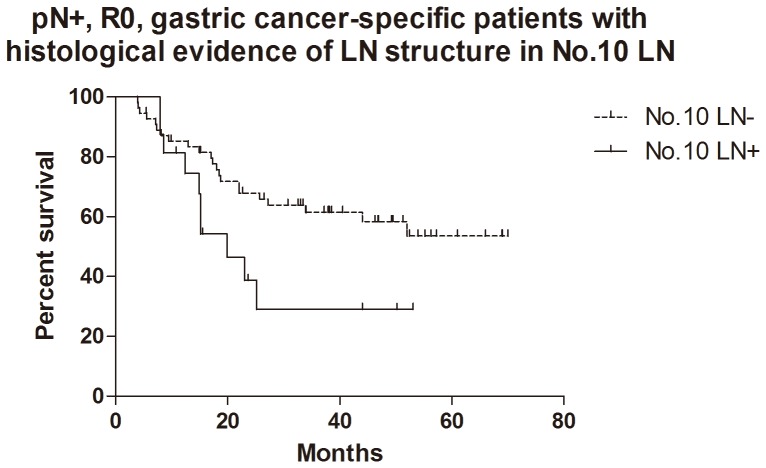
Kaplan-Meier survival analysis of pN+ stage gastric cancer specific + R0 resection group in patients with histological evidence of LNs structure in No.10 LNs.

For the patients with histological evidence of LNs structure in No.10 LNs, the 1- and 2-year cumulative survival rates of overall survival, GC specific group, R0 resection group and GC specific + R0 resection group were 81% and 62%, 83% and 65%, 81% and 62%, 85% and 67% respectively (Table 3). Kaplan-Meier analysis also showed that the differences between negative and positive No.10 LNs were significant in overall survival (n = 94, p = 0.017, HR = 0.36, 95%CI [0.15–0.83],Figure 5), R0 resection group (n = 89, p = 0.010, HR = 0.30, 95%CI [0.12–0.74], Figure 6) and GC specific group (n = 94, p = 0.015, HR = 0.33, 95%CI [0.13–0.80], Figure 7). However, the difference was not significant in Cox regression (p = 0.082). Furthermore, in view of positive No.10 LNs likely present in progressive stages of the disease, we also conducted stratification analyses in pT4 stage, pN+ stage, pN2-3 stage and pTNM IIIC-IV stage. In GC specific + R0 resection group of all patients, Kaplan-Meier showed that the differences between negative and positive No.10 LNs were significant in pT4 stage (p = 0.029, Figure 8), pN+ stage (p = 0.026, Figure 9) and pN2-3 stage (p = 0.046, Figure 10), but not in pTNM IIIC-IV stage (p = 0.171). In GC specific + R0 resection group of patients with histological evidence of LNs structure in No.10 LNs, Kaplan-Meier showed that the differences between negative and positive No.10 LNs were significant in pT4 stage (p = 0.027, Figure 11) and pN+ stage (p = 0.043, Figure 12), but not in pN2-3 stage (p = 0.063)and pTNM IIIC-IV stage (p = 0.086).

### Complications and Mortality

Twenty-seven patients (27/205, 13.2%) suffered from postoperative complications, with surgery-related complications in 12 patients (12/205, 5.9%) and non-surgery-related complications in 16 patients (16/205, 7.8%). One patient underwent secondary operation because of intraperitoneal bleeding. Details of postoperative complications were shown in Table 4. Two patients (2/205, 0.98%) died caused by massive gastrointestinal bleeding and type II respiratory failure respectively within one month after operation.

**Table 4:** Details of postoperative complications.

Complications	N = 205	%
Pancreatic fistula	1	0.49%
Lymphatic chyle leakage	1	0.49%
Digestive tract bleeding	2	0.98%
Intraperitoneal hemorrhage	1	0.49%
Intraperitoneal infection	1	0.91%
Wound infection	1	0.49%
Gastroparesis	2	0.98%
Hiccup	2	0.98%
Vomiting	1	0.49%
Diarrhea	1	0.49%
Pulmonary infection	13	6.3%
Respiratory failure	1	0.49%
Bacteremia	1	0.49%
Delirium	1	0.49%
Overall	27	13.2%

## Discussion

In this retrospective study, we calculated the metastatic rates of No.10 LNs in different subgroups and analyzed the correlated factors of No.10 LNs metastasis. The metastatic rates of No.10 LNs were 8.8% in all patients and 18.4% in patients with histological evidence of No.10 LNs, which were between 7.3%–30% as reported in previous researches [10]–[17]. Another metastatic-related characteristic, we need to notice, was the metastatic degree. The definition of metastatic degree of a certain station LNs was the ratio between the number of metastatic LNs and the total number of harvested LNs. However, the metastatic degree is reported rare, of which the significance is not very clear. From our study, the metastatic degree of No.10 LNs was 23.0% (47/204) that is higher than the result from a domestic study [29].

In this study, we also analyzed the metastatic rates of No.10 LNs at different locations of stomach. Longitudinally, carcinomas at UM/MU or UML of stomach had remarkably higher metastatic rates of No.10 LNs than other locations. At the same time, the metastatic rates of No.10 LNs were also significantly higher when tumors were located at the greater curvature, posterior wall or multi-walls of stomach, which was similar to the results of some previous reports [21], [29]. It was rational that these locations associated with higher metastatic rates of No.10 LNs according to the lymphatic drainage principle. However, some study found no obvious difference among tumor locations [17]. In this study, we also calculated the metastatic rates of No.10 LNs in proximal (6.5%, 14.3%) and total (9.4%, 19.5%) gastrectomy of all patients and patients with histological evidence of LNs structure in No.10 LNs. For the proximal GC, the principal surgery was total gastrectomy in our department to achieve better postoperative quality of life [30]. Proximal gastrectomy was predominantly performed in patients with smaller tumor. Therefore, the metastatic rate of No.10 LNs was lower in proximal gastrectomy than in total gastrectomy.

Besides the tumor location, tumor size was also significantly associated with the metastatic rate of No.10 LNs. The maximal diameter of tumors larger than 5 cm indicated a remarkably increasing metastatic rate of No.10 LNs, which was similar to the previous studies [14], [17], [19]. With respect to pTNM stage, positive No.10 LNs mainly appeared in the tumors with advanced stage, as similarly reported in other researches [14], [17], [19], [26]. In addition, we also focused on the relationship among No.10 LNs and other LNs, which was reported rarely. The univariate analysis demonstrated that many other regional LNs within D2 range had the relevance to the metastasis of No.10 LNs, even including some LNs (No.1, 2, 3, 6, 12a LNs) distal to No.10 LNs. This result could imply the intricate interactions among regional LNs to some extent. Some studies showed No.4sb LNs, No.4sa LNs and No.11 LNs were significant correlated with positive No.10 LNs [21], [29]. However, in our research, the multivariate analysis through logistic regression confirmed that pN stage and No.4sa LNs were independent risk factors of metastatic No.10 LNs.

No.10 LNs are located at splenic hilum, distributing closely along splenic hilar vessels and adjacent to No.4sa LNs No.4sb LNs and No.11d LNs as well. As we know, No.4sa LNs are located along the short gastric vessels and No.4sb LNs along left gastroepiploic vessels, which are continuation of the splenic vessels. It is known that lymphatics are the main metastatic routes for tumor cells and that the direction of lymphatic drainage of upper left stomach is from the stomach to the spleen. Tumor cells from the primary lesion in the stomach enter into lymphatics and then reach the splenic hilar area, which means tumor cells arrive at No.10 LNs. Then the lymphatic drainage flows along splenic vessels to the celiac artery. The univariate analysis showed the close relationship among No.10 LNs, No. 4sa LNs, No. 4sb LNs, No. 11d LNs and No. 9 LNs. Nonetheless, the logistic regression only confirmed the significance of No.4sa LNs in the metastasis of No.10 LNs.

The relationship between positive No.10 LNs and prognostic outcome is still controversial, though more studies showed poor postoperative survival rate [19]–[21]. In some study, No.10 LNs were even recommended as group N3, because the poor prognosis of the metastatic No.10 LNs was similar to that of positive para-aortic LNs [14]. In our research, Kaplan-Meier analysis indicated that metastatic No.10 LNs caused significantly worse survival outcomes than negative group in all patients (p = 0.006), R0 group (p = 0.003), GC specific group (p = 0.007) and GC specific + R0 resection group (p = 0.003) in all patients, as well as all patients with histological evidence of LNs structure in No.10 LNs (p = 0.017), R0 group (p = 0.010), GC specific group (p = 0.015) and GC specific + R0 resection group (p = 0.009) in patients with histological evidence of LNs structure in No.10 LNs. Moreover, we also did the stratification analyses to find out the prognostic significance of No.10 LNs. The results showed that the differences between negative and positive No.10 LNs were significant in pT4 stage (p = 0.029), pN+ stage (p = 0.026) and pN2-3 stage (p = 0.046) GC specific + R0 resection group of all patients, as well as pT4 stage (p = 0.027) and pN+ stage (p = 0.043) GC specific + R0 resection group of patients with histological evidence of LNs structure in No.10 LNs. Although the differences were not significant in pTNM IIIC-IV stratification, the trends were emerging. Through Cox regression analysis, we only found that No.10 LNs was an independent prognostic factors in R0 group in all patients (p = 0.031). These results were similar to other previous studies [14], [17]–[19]. Although no significant difference was found in other groups through Cox regression, we still thought that metastatic No.10 LNs was an important indicator of prognosis, because positive No.10 LNs usually originated from more advanced pathological stage and larger sized tumors, and led to poor prognosis. In the meantime, more high quality prospective randomized controlled trials with long-term follow-up are still necessary to assess the clinical value of No.10 LNs.

For the sake of close relationship between No.10 LNs and spleen, it is controversial whether to perform the splenectomy. From the previous studies, gastrectomy combined with conventional splenectomy did not bring the benefit for survival but cause more morbidity [8], [27]. Additionally, because of the poor prognosis of metastatic No.10 LNs, the effectiveness of prophylactic splenectomy was indeed uncertain [17]. In our practice, prophylactic splenectomy was not performed. However, if spleen was invaded by GC or No.10 LNs were confirmed as metastasis, gastrectomy combined with splenectomy was indicated to perform [27].

There were still some limitations of this study. The histological evidence of LNs structure in No.10 LNs was not demonstrated in approximate half patients. One of the reasons why so many fat tissues were harvested might be due to anatomic variation. To eliminate the limitation in this study, we did subgroup analysis in patients with histological evidence of LNs structure in No.10 LNs to calculate the relative factor. And the results were similar to all patients. Another limitation was that there were proximal and total gastrectomies, laparoscopy-assisted and open approaches in this study. However, proximal and laparoscopy-assisted gastrectomies were mainly indicated for small tumors and the differences between proximal and total, laparoscopy-assisted and open gastrectomies were focused on the quality of life. Therefore, the survival outcome was not influenced very much by different surgical procedures.

## Conclusion

In summary, although the metastatic rate of No.10 LNs was 8.8%, dissection of No.10 LNs still had clinical value due to the poor prognosis in positive cases and the close relationship with TNM stage, tumor location and size. Additionally, attentions should be also paid to its correlated factors including pN stage and No.4sa LNs.
